# Monitoring COVID-19 occurrence in a resource-limited setting – COVID-19 sentinel surveillance in Malawi

**DOI:** 10.1371/journal.pgph.0004158

**Published:** 2026-01-08

**Authors:** Godwin Ulaya, Alinune Kabaghe, Christel Saussier, Ellen MacLachlan, Joshua Smith-Sreen, Chaplain Katumbi, George Bello, Terence Tafatatha, Limbikani Chaponda, Bernard Mvula, Matthews Kagoli, Benson Chilima, Joseph Bitilinyu- Bangoh, Laphiod Chisuwo, Yusuf Babaye, Moses Chitenje, Barbara Bighignoli, Fred Bangara, Ireen Namakhoma, Annie Chauma-Mwale, Gabrielle O’Malley, Kelsey Mirkovic, Nellie Wadonda-Kabondo

**Affiliations:** 1 International Training and Education Center for Health (I-TECH) Malawi, University of Washington, Lilongwe, Malawi; 2 Division of HIV and TB, US Centers for Disease Control and Prevention Lilongwe, Lilongwe, Malawi; 3 International Training and Education Center for Health (I-TECH), University of Washington, Seattle, Washington, United States of America; 4 Alpert Medical School of Brown University, Providence, Rhode Island, United States of America; 5 Public Health Institute of Malawi (PHIM), Lilongwe, Malawi; 6 Department of Rare Disease, Amgen Biotechnology Research, Minneapolis, Minnesota, United States of America; PLOS: Public Library of Science, UNITED STATES OF AMERICA

## Abstract

The routine COVID-19 surveillance in Malawi that relied on retrospective reporting could not efficiently steer timely measures to the rapidly evolving pandemic. To monitor real-time changes in infections and inform the COVID-19 response, we implemented an active sentinel surveillance system from July to December 2022. Symptomatic and asymptomatic patients with SARS-CoV-2 in selected health facilities (HFs) and anyone aged ≥5 years entering at Point of Entry (PoEs) sites were eligible to participate. Self-reported epidemiological and clinical data, and nasopharyngeal specimens were collected from 9,305 participants. A higher overall SARS-CoV-2 RT-PCR positivity rate was observed at HFs, 8.9% among symptomatic and 6.5% among asymptomatic patients, versus 3.5% at PoEs. The positivity trends among symptomatic and asymptomatic patient groups showed a similar pattern throughout the period. This active surveillance complemented routine surveillance, especially during a low incidence period and highlighted the need to target both symptomatic and asymptomatic populations.

## Introduction

SARS-CoV-2 infection was first confirmed in Malawi on 2nd April 2020 [1]. The Government of Malawi subsequently activated a national Public Health Emergency Operations Center to coordinate the multisectoral response to the COVID-19 pandemic [2]. Despite the government’s initial response within the capacity of its resources, the surge of cases in the communities and the porous Points of Entry (PoEs) posed challenges to track changes in the incidence of COVID-19. Additionally, the limited investment in and prioritization of public health programs contributed to inadequate capacity in some health facilities (HFs) to routinely test for SARS-CoV-2. As a result, there was a reduced ability to detect cases and monitor changes in real-time [3]. Although suspected COVID-19 cases were confirmed using both rapid diagnostic and polymerase chain reaction (PCR) reaction testing and reported, surveillance was passive and circulating variants were not systematically tracked. The testing algorithm used in Malawi was restricted to those that were symptomatic, despite evidence of transmissibility of laboratory-confirmed SARS-CoV-2 infections among asymptomatic individuals [4]. Access barriers to HFs, particularly in rural areas, further weakened surveillance efforts by reducing or delaying case detection, slowing reporting, and limiting the effectiveness of public health intervention [5]. Challenges with data collection and reporting tools, and processes also affected the quality, timeliness, and accuracy of the available data, making it harder to monitor trends and respond promptly [6].

Although preventive and mitigation measures were recommended, periods of low reported COVID-19 incidence were accompanied with a noticeable general decline in adherence to these measures within communities and among healthcare workers. There was also relaxation in the overall routine surveillance system, with reduced testing and delayed reporting. Moreover, the emergence of the polio and cholera outbreaks in early 2022 compounded the situation and diverted attention and resources away from passive COVID-19 surveillance, further hindering the monitoring of the COVID-19 situation in Malawi [7,8].

In response to these challenges, the Public Health Institute of Malawi (PHIM), with support from its partners, established an active, real-time COVID-19 sentinel surveillance. This initiative was designed to complement routine surveillance by rapidly detecting changes in SARS-CoV-2 cases and allowing better characterization of SARS-CoV-2 [9]. The surveillance system objectives were to detect an increase in SARS-CoV-2 infections early, to characterize persons and locales at greater risk, and to identify possible reasons for increased risk. In this paper, we describe the trends in symptomatic and asymptomatic SARS-CoV-2 infections and patients’ demographic and behavioral characteristics during the first six months of the COVID-19 sentinel surveillance in Malawi.

## Methods

### Sentinel surveillance design

This surveillance initiative aimed at monitoring epidemiological and clinical trends in SARS-CoV-2 infection among individuals sampled at sentinel sites in Malawi. It was conducted as a pilot project, supported by partners, from July to December 2022. As a pilot initiative, the goal was to assess feasibility, with the plan for the Ministry of Health (MOH) to take over. The COVID-19 sentinel surveillance methodology was adapted from the WHO recommendations on integrated sentinel surveillance of influenza and SARS-CoV-2 [9].

### Sentinel surveillance locations

**Fig 1:**
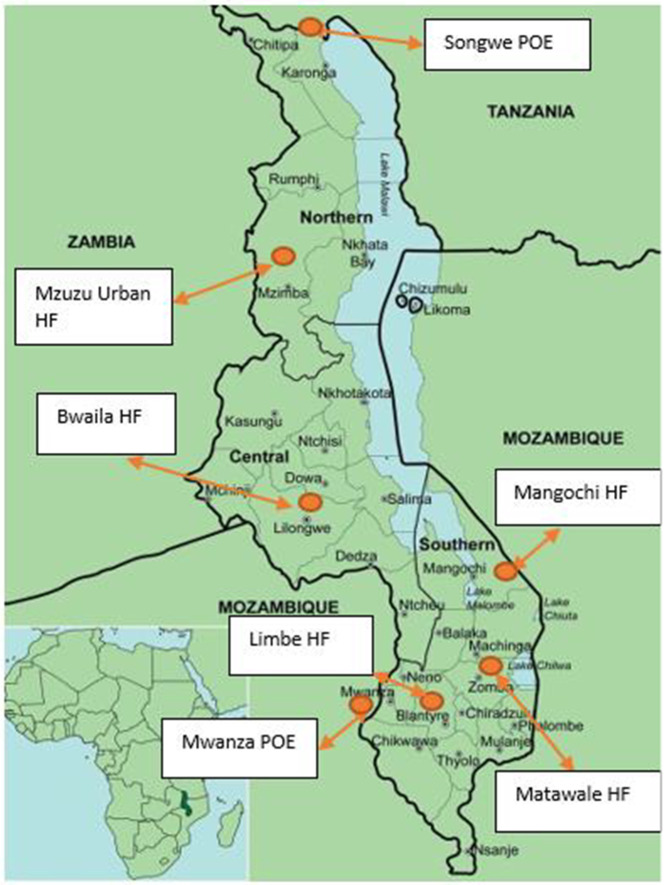
Map showing the seven COVID-19 sentinel sites across Malawi, July – December 2022[10]. HF: health facility, POE: point of entry.

The sentinel surveillance was set up in July 2022 in seven purposively selected sites in seven districts: five HFs— Limbe Health Centre (Blantyre), Bwaila Hospital (Lilongwe), Matawale Health Centre (Zomba), Mzuzu Urban Health Centre (Mzimba North), and Mangochi District Hospital (Mangochi)— and two border PoEs—Mwanza border (Mwanza) and Songwe border (Karonga) (Fig 1). The HFs were selected from the districts with the highest number of reported SARS-CoV-2 infections and from facilities with a high volume of patients [10]. The PoEs were selected due to their high-volume of incoming travelers from Mozambique and Tanzania, respectively.

### Study populations

In HFs, the study population consisted of patients of any age seeking healthcare for any reason. We excluded severely ill patients, those under 18 years old without a legal guardian, or those who had previously participated in the survey within 14 days. Eligible participants were screened through clinical assessment and categorized as having either SARS-CoV-2 symptomatic or asymptomatic infection based on the WHO integrated case definition for SARS-CoV-2 infection and Influenza. In line with WHO case definitions, symptomatic participants had either Influenza-like illness (ILI) defined as symptom onset within past 10 days, fever of 38^o^C or more and respiratory infection (cough) or acute respiratory illness (ARI) defined as at least one of cough, sore throat, shortness of breath, runny nose with or without fever and a clinician’s judgement that the illness is due to an infection. Participants classified as symptomatic met the criteria for ILI or ARI, while asymptomatic participants did not meet any of these definitions [9]. At the borders, persons ≥ 5 years of age entering the country were eligible to participate. In both groups, participants or their guardians were required to provide consent.

### Sample size and sampling technique

The HF sample size calculation assumed a SARS-CoV-2 infection prevalence of approximately 2% within the general HF population based on results from previous local COVID-19 surveys [11]. To ensure detection of at least one case of SARS-CoV-2 infection per week, given the 2% assumed prevalence and an estimated 10% refusal rate, each site aimed to recruit 50 symptomatic and 25 asymptomatic participants per week. This approach was consistent with WHO guidance on integrated SARS-CoV-2/Influenza surveillance [9].

Similarly, the sample size for the PoEs followed the same assumptions—a 2% infection prevalence and a 10% refusal rate— aiming to detect at least one SARS-CoV-2 case per week. Based on WHO technical guidance for SARS-CoV-2 surveillance, each PoE had an enrollment target of 50 travelers per week.

As part of ongoing government procedures at the time of the surveillance, everyone accessing the HFs compound were screened using an eligibility checklist and categorized as asymptomatic and symptomatic. This sentinel surveillance consecutively sampled the first ten consenting symptomatic and five asymptomatic participants during weekdays (Mondays to Fridays).

For PoE, each week a sampling interval was determined based on the estimated number of travelers crossing the border until a maximum of 50 participants were enrolled each week. A total of 10 travelers daily were selected from points of entry using systematic sampling, regardless of the presence of SARS-CoV-2 infection symptoms.

### Data and specimen collection

Before rolling out the implementation of the COVID-19 sentinel surveillance, all staff were trained on the protocol procedures. A dedicated surveillance assistant oversaw activities at each site and was responsible for data entry and quality control. Questions were administered using a structured questionnaire installed on an electronic tablet using the Open Data Kit (ODK) platform. The questionnaire was available in English, Chichewa, Tumbuka and KiSwahili. We collected self-reported information on socio-demographics, past exposure to COVID-19, current signs and symptoms of influenza-like illness, contact with PCR-confirmed SARS-CoV-2–infected persons, national and international travel, attendance to gatherings, adherence to COVID-19 preventive measures, underlying health conditions— HIV, cardiovascular disease, chronic respiratory disease, diabetes, and other medical conditions—, and vaccination against SARS-CoV-2.

A nasopharyngeal (NP) specimen was collected from all participants. The NP specimens were immediately put in viral transport media and transported in temperature regulated cooler boxes for storage at 2–8 °C before testing. NP specimen tests were done at the sentinel facilities utilizing RT-PCR GeneXpert platforms within 24 hours of collection. Specimens that tested positive for SARS-CoV-2 were stored in minus 80 degrees freezers within 72 hours of specimen collection prior to shipment to the PHIM national reference laboratory for genomic sequencing (Results of genomic sequencing will be presented separately). All test results were incorporated into the routine surveillance system. The district health offices where the cases were detected were responsible for communicating the test results to the participants.

### Data management and analysis

Data collected was daily sent through internet connection to a secure server to ensure timeliness. The data were downloaded from the server and imported into Stata software, version 14.2 for data cleaning and analysis [12]. The dataset was and is still being stored at the Birth Defect Surveillance Server which is under the Public Health Institute of Malawi (PHIM). In terms of data quality, each participant was assigned a unique identifier upon enrollment for linking ODK data and laboratory results. If values exceeded clinically expected ranges, queries were sent to the concerned study site to confirm their validity. Data from all the sentinel sites were assessed for consistency on a weekly basis. Data completeness was assessed by checking the weekly enrolment targets against the actual weekly enrolments. Outputs tables and graphs including weekly and cumulative enrolment figures, collected specimens, PCR results, and vaccination status for all participants were generated on a weekly basis and shared with the PHIM for investigation and action. COVID-19 vaccination status was self-reported by participants. Participants who reported two doses of COVID-19 AstraZeneca or Pfizer vaccine or one dose of COVID-19 Johnson & Johnson vaccine were categorized as “fully vaccinated” while those receiving one dose of COVID-19 AstraZeneca or Pfizer vaccine were categorized as “partially vaccinated”. Those without any dose of any vaccine were categorized as “not vaccinated at all”.

We conducted descriptive [frequencies, medians, and interquartile ranges (IQRs)] and bivariate analyses (not included in this manuscript) of sociodemographic and clinical factors and associations with testing positive by RT-PCR. We also compared (PRs) between symptomatic participants, asymptomatic participants, and travelers. Multivariable logistic regression (with a binary outcome: positive vs negative PCR result) analyses assessed associations between testing positive and various independent variables. The independent variables were sociodemographic (age, sex, education level, occupation and location based on health facility or point of entry) and clinical factors (vaccination status and underlying factors).. We adjusted for sex, age and education variables. Selection of the covariates was based on clinical and epidemiological importance as well as statistical importance based on bivariate analyses (chi-square). Crude and adjusted odds ratios (aOR) and 95% confidence intervals (CIs) were calculated. We considered a P value ≤0.05 to be statistically significant.

### Ethical considerations

Ethics approvals were granted by the National Health Services Research Council (Reference NHSRC # 22/04/2896) and the protocol was reviewed by CDC, deemed not research, and was conducted consistent with applicable federal law and CDC policy [13,14].

All participants gave their written informed consent or assent prior to their participation in the surveillance procedures. Informed consent from a guardian was required for all children under 18 years of age, except for emancipated minors.

## Results

### Participant screening and enrollment

**Fig 2:**
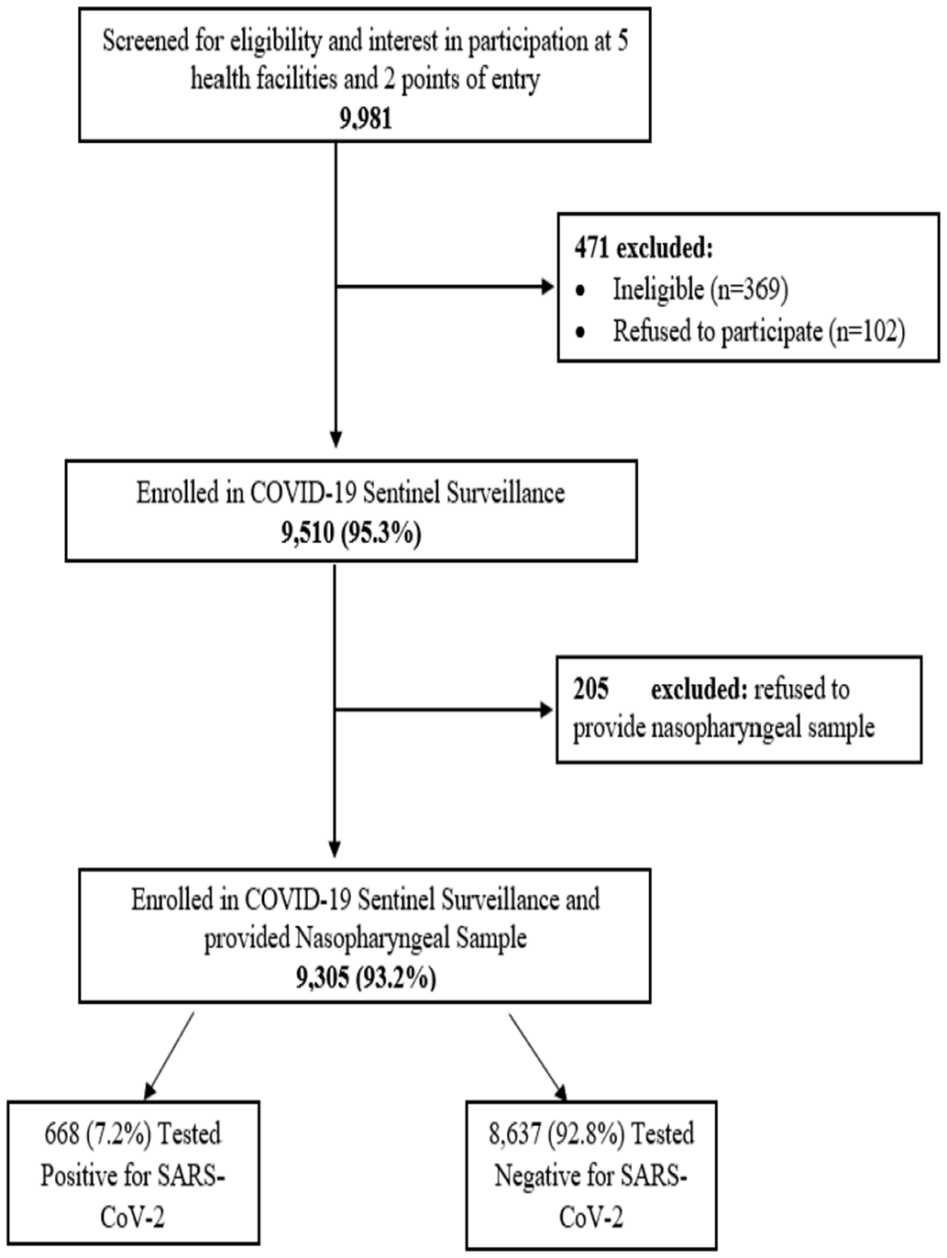
Flow diagram of participants inclusion, COVID-19 sentinel surveillance, Malawi, July–December 2022.

A total of 9,981 individuals were screened across all seven sentinel sites. Among them, 4.7% (n = 471) were excluded from the study, either because they refused to give consent/assent (n = 102) or were ineligible based on surveillance exclusion criteria (n = 369). A total of 9,510 participants (95.3%) were enrolled;205 declined to provide NP specimens and were excluded. A total of 9,305 participants (93.2%) provided both the NP specimens and responded to the questionnaire (Fig 2).

### Participant characteristics by sentinel surveillance site type and symptom status

Table 1 provides a summary of participant characteristics categorized by type of sentinel surveillance site and symptom status. In HFs, fewer participants were enrolled at Mzuzu and Limbe health centers (15.6% and 15.0% respectively). More participants were enrolled at Mwanza PoE (59.5%) compared to Songwe PoE (40.5%). Most of the respondents enrolled at the health facilities were aged between 15–29 years (45.9% among symptomatic and 43.9% among asymptomatic) and most enrolled travelers were aged 30–49 years (59.0%). At HFs, females represented 61.1% and 61.9% of asymptomatic and symptomatic groups, respectively. Among travelers, 70.7% were males. Most of the respondents had attended secondary education in both the HFs (72.7%) and PoEs (76.0%).

**Table 1:** Characteristics of enrolled participants by symptom status and type of sentinel surveillance site), COVID-19 sentinel surveillance, Malawi, July-December 2022.

		Patients at health facilities		Travelers at points of entry (POEs) N = 1735	Total study population N = 9305
		Symptomatic N = 4880	Asymptomatic N = 2690		
**Characteristic**		**n (%)**	**n (%)**	**n (%)**	**n (%)**
**Sentinel Site**					
Health Facilities	Mzuzu Urban	1033 (21.2)	150 (5.6)	NA	1183 (15.6)
	Bwaila	1096 (22.5)	655 (24.3)	NA	1751 (23.1)
	Mangochi	1099 (22.5)	740 (27.5)	NA	1839 (24.3)
	Matawale	993 (20.3)	670 (24.9)	NA	1663 (22.0)
	Limbe	659 (13.5)	475 (17.7)	NA	1134(15.0)
POEs	Songwe	NA	NA	703 (40.5)	703 (40.5)
	Mwanza	NA	NA	1032 (59.5)	1032 (59.5)
**Age (years)**	0-14	236 (4.8)	102 (3.8)	41 (2.4)	379 (4.1)
	15-29	2240 (45.9)	1183 (43.9)	514 (29.6)	3937 (42.3)
	30-49	1858 (38.1)	1104 (41.0)	1024 (59.0)	3986 (42.8)
	≥50	546 (11.2)	301 (11.2)	156 (9.0)	1003 (10.8)
Median, years (IQR)		29 (17)	30 (16)	34 (13)	31 (17)
**Sex**	Female	2981 (61.1)	1664 (61.9)	509 (29.3)	5154 (55.4)
	Male	1899 (38.9)	1026 (38.1)	1226 (70.7)	4151 (44.6)
**Highest level of education**	None	316 (6.5)	117 (4.4)	11(0.6)	444 (4.8)
	Primary	1089 (22.3)	541 (20.1)	405 (23.3)	2035 (21.9)
	Secondary	3475 (71.2)	2032 (75.5)	1319 (76.0)	6826 (73.3)
**Occupation**	In school	706 (14.5)	282 (10.5)	137 (7.9)	1125 (12.1)
	Employed/Business	1568 (32.1)	793 (29.5)	806 (46.5)	3167 (34.0)
	Farmer	690 (14.1)	358 (13.3)	89 (5.1)	1137 (12.2)
	Unemployed	1916 (39.3)	1257 (46.7)	703 (40.5)	3876 (41.7)
**Vaccination Status**	Non-Vaccinated	3631 (74.4)	2070 (77.0)	1220 (70.3)	6921 (74.4)
	Partially vaccinated	303 (6.2)	192 (7.1)	199 (11.5)	694 (7.5)
	Full vaccination	946 (19.4)	428 (19.9)	316 (18.2)	1690 (18.1)
**Underlying Conditions**	HIV	259 (5.3)	290 (10.8)	81 (4.7)	630 (6.8)
	CV diseases*	180 (3.7)	66 (2.5)	42 (2.4)	288 (3.1)
	Chronic respiratory disease^†^	204 (4.2)	35 (1.3)	21 (1.2)	260 (2.8)
	Diabetes	30 (0.6)	18 (0.7)	10 (0.6)	58 (0.6)
	Others	127 (2.6)	80 (3.0)	33 (1.9)	240 (2.6)
	None	4080 (83.6)	2201 (81.8)	1548 (89.2)	7829 (84.1)

*CV diseases: chronic cardiac disease and/or hypertension.

^†^Chronic respiratory disease: including asthma and tuberculosis.

In the HFs, most of the respondents in both symptomatic and asymptomatic groups were “unemployed” (39.3% and 46.7% respectively). At PoEs, most participants were either employed or in business (46.5%).

About three quarters of the respondents in each category were not vaccinated:74.4% of symptomatic patients, 77.0% of asymptomatic patients and 70.3% of travelers. Less than one fifth in each category were fully vaccinated: 19.4% symptomatic, 19.9% asymptomatic and 18.1% of travelers.

Most respondents reported no underlying health condition (83.6%). Among the self-reported underlying conditions, HIV was the most reported: 5.3% of symptomatic patients, 10.8% of asymptomatic and 4.7% of travelers.

### SARS-CoV-2 positivity rate trends among health facility respondents and travelers

**Fig 3:**
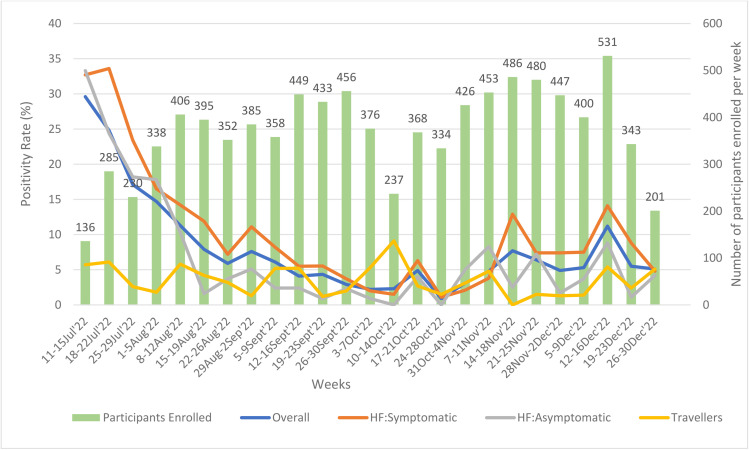
Trends of cumulative COVID-19 positivity rate, overall, among symptomatic and asymptomatic, and travelers, COVID-19 sentinel surveillance, Malawi, July-December 2022.

Fig 3 shows trends of SARS-CoV-2 positivity rate in those recruited at the HFs and the travelers. The highest overall positivity rate of 29.6% was recorded in the week of 11-15 July 2022 when the lowest number of participants were recruited (n = 136) while the lowest positivity of 0.9% was recorded in the week of 24-28 October 2022 (n = 334). The average overall positivity rate was 8.0%. The highest number of participants recruited was 531 in the week 12-16 December 2022. On average 372 participants were recruited every week. Throughout the sentinel surveillance period, the positivity trends among the symptomatic and asymptomatic patients showed a similar pattern, with the symptomatic group showing slightly higher rates in most of the time points.

### Association of SARS-CoV-2 positivity with demographics and behavioral factors

Tables 2 and 3 presents the association of SARS-CoV-2 positivity rate with various demographic and behavioral factors categorized by symptomatic patients, asymptomatic patients, and travelers. The results of unadjusted results are only presented in the table. Below is a description of adjusted model results.

**Table 2:** Association of SARS-CoV-2 positivity rates with demographic and behavioral factors among symptomatic patients, COVID-19 sentinel surveillance, Malawi, July-December 2022.

Characteristics	Symptomatic patients		
	n = 4880		
	Percent positivity (95% CI)	Unadjusted OR (95% CI)	Adjusted* OR (95% CI)
Overall SARS-CoV-2 positivity rate (%)	8.9 (8.1-9.7)	NA	NA
Sex			
Male	8.2 (6.9-9.4)	Reference	
Female	9.3 (8.3-10.4)	1.2 (0.9-1.4)	1.2 (0.9-1.5)
Age in years			
0-14^‡^	8.8 (5.5-13.2)	Reference	
15-24	9.1 (7.9-10.3)	1.0 (0.6-1.6)	0.9 (0.6-1.6)
25-49	8.3 (7.1-9.6)	0.9 (0.6-1.5)	0.8 (0.5-1.4)
50+	9.7 (7.3-12.5)	1.1 (0.6-1.9)	0.9 (0.6-1.8)
Education			
None	13.9 (10.3-18.2)	Reference	
Primary	9.6 (2.0-25.7)	0.7 (0.2-2.3)	1.0 (0.7-1.5)
Secondary	8.5 (7.7-9.3)	0.6 (0.4-0.8)	0.8 (0.5-1.1)
Occupation			
Unemployed/Retired	6.9 (5.8-8.1)	Reference	Reference
Student	10.5 (8.3-12.9)	1.5 (1.2-2.1)	1.1 (0.8-1.6)
Employed/Business	10.8 (9.3-12.4)	1.6 (1.3-2.1)	1.3 (1.0-1.7)
Farmer	8.5 (6.4-10.7)	1.2 (0.9-1.7)	1.4 (1.0-1.9)
Vaccination status^†^			
Unvaccinated	7.9 (7.0-8.8)	Reference	Reference
Partially vaccinated	13.2 (9.6-17.5)	1.8 (1.2-2.5)	1.7 (1.2-2.4)
Fully vaccinated	11.1 (9.1-13.2)	1.4 (1.1-1.8)	1.1 (0.9-1.5)
Any Underlying?			
No	8.4 (7.6-9.4)	Reference	Reference
Yes	8.1 (4.9-12.4)	1.3 (1.0-1.7)	1.6 (0.9-2.7)
HIV			
No	8.9 (8.1-9.80	Reference	Reference
Yes	8.2 (5.1-12.2)	0.9 (0.5-1.4)	0.6 (0.3-1.1)
CVD			
No	8.7 (7.9-9.5)	Reference	Reference
Yes	13.3 (8.7-19.1)	1.5 (0.9-2.3)	0.9 (0.5-1.8)
CRD			
No	8.8 (8.0-9.6)	Reference	Reference
Yes	10.8 (6.9-15.9)	1.2 (0.8-1.9)	0.8 (0.4-1.5)
Diabetes			
No	8.9 (8.1-9.7)	Reference	Reference
Yes	10.0 (2.1-26.5)	1.1 (0.3-3.7)	0.8 (0.2-2.7)
Health Facility			
Bwaila	83.(7.0-9.4)	Reference	Reference
Mzuzu Urban	19.5 (16.8-20.9)	4.1 (3.0-5.5)	3.9 (2.7-5.4)
Mangochi	7.7 (5.9-8.2)	1.6 (1.1-2.2)	1.6 (1.1-2.3)
Matawale	4.1 (3.2-4.9)	0.8 (0.5-1.2)	0.8 (0.5-1.2)
Limbe	7.2 (6.1-9.0)	1.3 (0.9-2.0)	1.3 (0.9-2.0)

**Table 3:** Association of SARS-CoV-2 positivity rates with demographic and behavioral factors among Asymptomatic patients and Travelers, COVID-19 sentinel surveillance, Malawi, July-December 2022.

Characteristics	Asymptomatic patients			Travellers at POEs		
	n = 2690			n = 1735		
	Percent positivity (95% CI)	Unadjusted OR (95% CI)	Adjusted* OR (95% CI)	Percent positivity (95% CI)	Unadjusted OR (95% CI)	Adjusted* OR (95% CI)
Overall SARS-CoV-2 positivity rate (%)	6.5 (5.6-7.5)	NA	NA	3.5 (2.6-4.4)	NA	NA
Sex						
Male	7.2 (5.7-8.9)	Reference		3.3 (2.3-4.4)	Reference	
Female	6.1 (4.9-7.3)	0.8 (0.6-1.1)	0.9 (0.6-1.3)	3.9 (2.4-6.0)	1.2 (0.7-2.1)	1.0 (0.6-1.8)
Age in years						
0-14^‡^	6.9 (2.8-13.6)	Reference		4.9 (0.5-16.5)	Reference	
15-24	6.0 (4.7-7.5)	0.9 (0.4-1.9)	0.9 (0.4-2.1)	3.7 (2.2-5.7)	0.9 (0.6-1.4)	0.9 (0.2-4.7)
25-49	6.8 (5.4-8.4)	0.9 (0.4-2.2)	1.0 (4.3-2.4)	3.6 (2.5-4.9)	0.8 (0.6-1.2)	0.9 (0.2-4.7)
50+	7.3 (4.6-10.8)	1.1 (0.4- 2.5)	1.2 90.5-2.9)	1.3 (0.2-4.5)	0.9 (0.6-1.5)	0.3 (0.1-2.3)
Education						
None	4.3 (1.4-9.6)	Reference		0	Reference	
Primary	15.4 (1.9-45.4)	4.1 (0.7-23.5)	2.6 (0.9-7.0)	8.3 (0.2-38.5)	NA	NA
Secondary	6.6 (5.6-7.6)	1.6 (0.6-3.9)	1.6 (0.6-4.1)	3.5 (2.7-4.5)	NA	NA
Occupation						
Unemployed/Retired	5.9 (4.7-7.3)	Reference	Reference	3.6 (2.3-5.2)	Reference	Reference
Student	7.4 (4.7-11.1)	1.3 (0.7-2.1)	0.9 (0.5-1.5)	8.0 (4.1-13.9)	2.4 (1.1-4.9)	2.5 (0.9-6.5)
Employed/Business	8.3 (6.5-10.5)	1.5 (1.0-2.0)	1.1 (0.7-1.7)	2.6 (1.6-3.9)	0.7 (0.4-1.3)	0.6 (0.2-1.5)
Farmer	3.9(2.2-6.5)	0.7 (0.4-1.2)	0.7 (0.4-1.2)	3.4 (0.7-9.5)	0.9 (0.3-3.2)	0.9 (0.2-2.9)
Vaccination status^†^						
Unvaccinated	6.3 (5.2-7.4)	Reference	Reference	3.1 (0.1-3.5)	Reference	Reference
Partially vaccinated	5.7 (2.8-10.0)	0.9 (0.5-1.7)	0.8 (0.4-1.5)	1.0 (3.9-9.6)	0.3 (0.1-1.3)	0.4 (0.1-1.6)
Fully vaccinated	7.9 (5.5-10.9)	1.3 (0.9-1.9)	1.2 (0.8-1.8)	6.3 (4.2-8.2)	2.1 (1.2-3.7)	2.3 (1.3-4.2)
Any Underlying?						
No	6.3 (5.3-7.4)	Reference	Reference	3.5 (2.6-4.6)	Reference	Reference
Yes	7.4 (5.3-10.1)	1.3 (0.9-1.9)	2.0 (1.0-4.0)	2.8 (0.9-6.4)	0.8 (0.3-1.9)	0.3 (0.1-4.0)
HIV						
No	6.5 (5.5-7.5)	Reference	Reference	3.5 (2.7-4.5)	Reference	Reference
Yes	6.9 (4.3-10.5)	1.2 (0.8-1.9)	0.5 (0.2-1.2)	2.5 (0.3-8.6)	0.7 (0.2-2.9)	1.8 (0.1-28.6)
CVD						
No	6.5 (5.6-7.5)	Reference	Reference	3.4 (2.6-4.4)	Reference	Reference
Yes	7.6 (2.5-16.8)	1.4 (0.6-3.1)	0.5 (0.2-1.6)	4.8 (0.5-16.1)	1.4 (0.3-5.9)	3.7 (0.3-48.9)
CRD						
No	6.6 (5.7-7.6)	Reference	Reference	3.4 (2.6-4.4)	Reference	Reference
Yes	2.9 (0.1-14.9)	0.8 (0.2-3.4)	0.2 (0.1-1.8)	4.7 (0.123.8)	1.4 (0.2-10.6)	3.2 (0.2-56.7)
Diabetes						
No	6.6 (5.6-7.5)	Reference	Reference	3.5 (2.7-4.5)	Reference	Reference
Yes	0	NA	NA	0	NA	NA
Health Facility						
Bwaila	8.0 (6.2-10.2)	Reference	Reference	NA	NA	NA
Mzuzu Urban	13.9 (9.3-19.7)	3.2 (1.8-5.5)	3.4 (1.9-6.1)	NA	NA	NA
Mangochi	6.2 (4.7-7.9)	1.2 (0.8 – 1.9)	1.4 (0.9-2.2)	NA	NA	NA
Matawale	3.9 (2.7-5.5)	0.8 (0.5-1.4)	0.9 (0.6-1.5)	NA	NA	NA
Limbe	7.9 (5.7-10.5)	1.2 (0.7-1.9)	1.0 (0.6-1.7)	NA	NA	NA
PoEs						
Mwanza	NA	NA	NA	3.4 (2.4-4.7)	Reference	Reference
Songwe	NA	NA	NA	3.4 (2.2-4.9)	0.9 (0.5-1.5)	0.9 (0.5-2.0)

*Variables for adjustment: sex, age, education.

^†^The first age category for travelers is 5–14.

^‡^Fully vaccinated = 2 doses of COVID-19 AstraZeneca or Pfizer vaccine or 1 dose of COVID-19 Johnson & Johnson; Partially = 1 dose of COVID-19 AstraZeneca or Pfizer vaccine; Unvaccinated = no vaccine dose.

^§^Weakened immune system = HIV status (Yes = HIV positive, No = HIV negative).

### Symptomatic patients

After adjusting for all potential confounders, partial vaccination was associated with higher SARS-CoV2 positivity compared with no vaccination (aOR: 1.7; 95% CI: 1.2-2.4). SARS-CoV-2 positivity was also higher among participants enrolled at Mzuzu Urban (aOR:3.9; 95% CI:2.7-5.4) and Mangochi (aOR: 1.6 95% CI: 1.1-2.3) health facilities compared to those enrolled at the referenced facility

### Asymptomatic patients

In the adjusted model, SARS-CoV-2 positivity was also higher among participants enrolled at Mzuzu Urban health facility compared to those enrolled at the referenced facility (aOR: 3.4; 95% CI: 1.9-6.1).

### Travelers

In the adjusted model, Travelers who were fully vaccinated also had higher SARS-CoV-2 positivity rate compared with those that were not vaccinated (aOR: 2.3; 95% CI: 1.3-4.2).

## Discussion

This sentinel surveillance was among the initial steps toward implementing integrated SARS-CoV-2 and influenza surveillance in a resource-limited setting [9]. It provided an opportunity to retrain healthcare workers on the importance of continued active tracking of SARS-CoV-2 infections and helped routine surveillance become embedded amidst other priority programs and emerging outbreaks. This, in turn, contributed to health systems strengthening at various levels. The methodology offered the unique advantage of assessing two different surveillance approaches within the same health system as the country prepared to adopt a fully integrated model [9]. Although the influenza component was not fully integrated during this period, the ability to identify SARS-CoV-2 infections among both symptomatic and asymptomatic individuals using a combined SARS-CoV-2/influenza case definition indicated that integration would be feasible—and potentially efficient, particularly in resource-limited settings. In contrast, the routine surveillance system alone may have missed many asymptomatic cases, especially in Malawi, where SARS-CoV-2 testing was mostly directed at individuals presenting with symptoms [15].

The sentinel system also helped address gaps in routine testing. Reports from the sub-saharan countries such as Malawi, indicate that testing was limited across public health facilities, with some routine testing sites ceasing operations altogether [5,16]. This low uptake might have been compounded during low incidence periods which typically coincides with a relaxation of COVID-19 control measures, when preventive communication and enforcement tend to be deprioritized [17]. Additionally, attention was shifting toward other public health emergencies, particularly the nationwide cholera outbreak that began in March 2022 [7]. The above observations highlight the challenges of sustaining active sentinel surveillance during periods of low perceived threat, given high operational cost for human resources, logistics and laboratory capacity. Although alternative approaches, such as Event-Based surveillance (EBS) and digital monitoring of Internet searches or mobile browsing, can provide early, low-cost signals of disease activity in Malawi, low reporting coverage, uneven Internet access, and limited clinical specificity constrain their reliability [18]. Consequently, active sentinel surveillance remains essential for generating reliable, laboratory-confirmed data in resource-limited settings. A hybrid multi-modal approach, combining digital or EBS signals with targeted active sampling, may improve early detection while managing operational demands and could become increasingly feasible as connectivity and resources improve.

The observed differences in sex distribution between participants enrolled at health facilities (HFs) and points of entry (PoEs) may reflect known gender-related roles. Women often visit health facilities more frequently than men due to their roles in childbearing and caregiving. Conversely, men are typically more engaged in business-related travel, consistent with observed enrollment patterns at PoEs [19].

Higher positivity rates at the start of the surveillance period may reflect overestimation due to smaller sample sizes during the early stages [20]. After this phase, the epidemiological curve derived from this sentinel surveillance closely mirrored that of routine surveillance systems [21]. Notably, this curve remained relatively flat compared to the previous year, consistent with predictions by Jiang et al., who attributed such trends to the collective effect of systematic interventions, including widespread vaccinations and preventive strategies deployed across multiple countries [22].

The similar trends in positivity rates between symptomatic and asymptomatic participants underscore the importance of targeting both groups for testing and prevention. Asymptomatic cases can be identified through contact tracing, or by intentionally sampling asymptomatic individuals on a routine basis within an integrated surveillance platform [23].

This surveillance found associations between SARS-CoV-2 positivity and health facility locations.. Mangochi District Hospital and Mzuzu Urban Health Centre demonstrated higher SARS-CoV-2 positivity compared to Bwaila Urban in Lilongwe among the symptomatic patients. These differences may be influenced by district-level variations in socio-economic status, health-seeking behavior, and access to preventive services, shaped in part by the degree of urbanization [22,24]. These district-level disparities are critical for guiding targeted allocation of resources in low-resource settings.

Notably, SARS-CoV-2 positivity was higher among symptomatic HF participants who were partially vaccinated and among fully vaccinated travelers, compared to the unvaccinated. However, this association was not apparent in asymptomatic participants—whether partially or fully vaccinated—nor among partially vaccinated travelers. These inconsistencies should be interpreted cautiously due to several limitations. Firstly, vaccine status was self-reported and thus susceptible to recall and classification bias. Secondly, the sample size in vaccinated categories was small, which limits the precision of observed effect sizes. Therefore, to better understand these patterns, further investigations targeting vaccine effectiveness in different real-world scenarios are warranted. Nonetheless, it is important to affirm that the efficacy and effectiveness of SARS-CoV-2 vaccines—particularly for preventing symptomatic disease—are well-established in scientific literature [25,26]. To enhance future analyses, surveillance systems should be intentionally designed to also capture variables related to behavior, given the impact of behavioral factors on vaccine effectiveness. One such behavioral phenomenon to consider is the Peltzman Effect—where individuals might reduce adherence to preventive measures due to the perceived protection conferred by vaccination [27]. If present in the population, such behavioral compensation could paradoxically increase transmission among vaccinated individuals who neglect interventions.

This pilot surveillance had several limitations. Factors such as the use of observational study design, the use of consecutive sampling in health facilities, the targeted population in health facilities and the reliance on self-reported vaccination data may limit the generalizability of the findings. In health facilities, the originally proposed systematic sampling design was not feasible due to constraints observed during piloting. As a result, consecutive sampling was used, introducing potential selection bias. Across all sites, the use of self-reported vaccination data might have also introduced recall and misclassification bias. Additionally, the questionnaire lacked data on disease severity and behavioral aspects of vaccination, both of which are critical to understanding observed outcomes more deeply. Another key limitation is the short surveillance period (July–December 2022), which precludes analysis of seasonal variations in SARS-CoV-2 transmission. This constraint reflects the pilot nature of the study, which aimed to assess feasibility before full transition to the Ministry of Health management. Future surveillance under MOH oversight—including broader population sampling and longer implementation periods—will offer greater opportunity for robust epidemiological assessment and trend analysis over time.

## Conclusion

This COVID-19 active sentinel surveillance complemented routine passive surveillance and was instrumental in the low incidence period. The surveillance offered lessons to feed into a full integration approach of SARS-CoV-2 and Influenza surveillance in Malawi as per WHO recommendations. Nevertheless, the observed lower prevalence of SARS-CoV-2 and the relatively flat trend imply that some preventative measures and systems were in place and working. The ongoing detection of cases in both symptomatic and asymptomatic groups, however, underscores the important need of continuous COVID-19 surveillance in all populations. Moreover, the ongoing detection of infections among travelers, despite instituted COVID-19 travel restrictions, indicates the need for continued surveillance and provides an opportunity to detect novel variants coming into the country. The results also underscore the need for in-depth study of the interaction between SARS-CoV-2 vaccinations and behavioral adherence to preventative measures so that sensitization messages can be formulated according to context and evidence. Another area for future research could involve conducting a cost-benefit analysis of active sentinel surveillance compared to alternative approaches such as hybrid systems that combine traditional surveillance systems with digital tools to enhance early detection and optimize resource use. Such analysis would help the development of more sustainable surveillance strategies.
